# Microplastics alter the functioning of marine microbial ecosystems

**DOI:** 10.1002/ece3.70041

**Published:** 2024-11-14

**Authors:** Daniel Montoya, Eugenio Rastelli, Raffaella Casotti, Vincenzo Manna, Anna Chiara Trano, Cecilia Balestra, Chiara Santinelli, Maria Saggiomo, Clementina Sansone, Cinzia Corinaldesi, Jose M. Montoya, Christophe Brunet

**Affiliations:** ^1^ Basque Centre for Climate Change (BC3) Leioa Spain; ^2^ IKERBASQUE, Basque Foundation for Science Bilbao Spain; ^3^ Theoretical and Experimental Ecology Station, CNRS Moulis France; ^4^ Fano Marine Center, Stazione Zoologica “Anton Dohrn” Fano Italy; ^5^ Stazione Zoologica “Anton Dohrn” Naples Italy; ^6^ National Institute of Oceanography and Applied Geophysics—OGS Trieste Italy; ^7^ Istituto di Biofisica Consiglio Nazionale Delle Ricerche Sezione Pisa Italy; ^8^ Università Politecnica Delle Marche, Dipartimento di Scienze e Ingegneria Della Materia, dell'Ambiente Ed Urbanistica Ancona Italy

**Keywords:** bacteria, ecosystem functioning, microplastics, oceans, phytoplankton

## Abstract

Microplastics pervade ocean ecosystems. Despite their effects on individuals or populations are well documented, the consequences of microplastics on ecosystem functioning are still largely unknown. Here, we show how microplastics alter the structure and functioning of pelagic microbial ecosystems. Using experimental pelagic mesocosms, we found that microplastics indirectly affect marine productivity by changing the bacterial and phytoplankton assemblages. Specifically, the addition of microplastics increased phytoplankton biomass and shifted bacterial assemblages' composition. Such changes altered the interactions between heterotrophic and autotrophic microbes and the cycling of ammonia in the water column, which ultimately benefited photosynthetic efficiency. The effects of microplastics on marine productivity were consistent for different microplastic types. This study demonstrates that microplastics affect bacteria and phytoplankton communities and influence marine productivity, which ultimately alters the functioning of the whole ocean ecosystem.

## Graphical abstract

Although microplastics are increasingly considered a global environmental challenge, their effects on communities and ecosystems are not clear. By using experimental mesocosms, we report that microplastics are able to alter the composition of both bacteria and phytoplankton communities and the interactions between them. Changes induced by microplastics on microbial dynamics have potential impacts on the functioning and services provided by oceans, in particular on ocean productivity.

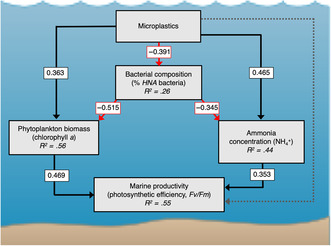

## 1 INTRODUCTION

The ubiquity, abundance, and persistence of microplastics in the environment make them a major challenge (Lavender‐Law, 2017). Plastic debris is present in marine, freshwater, and terrestrial systems, and their effects on the physiology of individual organisms are increasingly documented across trophic levels and taxonomic groups (GESAMP, 2016), including mammals (Besseling et al., 2015), crustaceans (Watts et al., 2014), fishes (Alomar & Deudero, 2017), and zooplankton (Cole et al., 2016). Microplastics ingestion can impair organisms' growth (Rochman et al., 2016), decrease fecundity rates (Sussarellu et al., 2016; Yokota et al., 2017), or feeding capacity (Corinaldesi et al., 2021), ultimately shortening organismal lifespan (Mao et al., 2018; Wright et al., 2013). However, major knowledge gaps still exist regarding the effects of microplastics at the community and ecosystem levels, especially within the planktonic environment. Given the primary role played by planktonic organisms in marine ecosystems, filling this knowledge gap is of paramount importance (Amaral‐Zettler et al., 2020; Jacquin et al., 2019).

Bacteria and phytoplankton can be considered the “engine” of the oceans as they dominate marine ecosystems in terms of both carbon fluxes and abundance, and modulate global ocean productivity and biogeochemical cycles (Nava & Leoni, 2021; Pomeroy et al., 2007). Therefore, any response of these organisms to environmental stressors may have cascading effects on the whole ocean ecosystem.

Bacteria are classified into two functional groups based on their nucleic acid content: high nucleic acid concentration (*HNA*) and low nucleic acid concentration (*LNA*) (Besmer et al., 2017; Bouvier et al., 2007; Gasol et al., 1999; Lebaron et al., 2001; Mao et al., 2022; Proctor et al., 2018). *LNA* and *HNA* functional groups have distinct metabolic and ecological functions (Hu et al., 2023; Song et al., 2019) and differ in their metabolic activity (Gasol & del Giorgio, 2000; Moran et al., 2015), ecophysiological requirements, and adaptative capacity (Pradeep Ram et al., 2020), which probably confers them different ecological roles (Liu et al., 2016). Whereas *HNA* bacteria are fast‐growing organisms with large genomes that make them more active (Gasol & del Giorgio, 2000) and thrive under high nutrient and carbon concentrations (Hu et al., 2020; Kaartokallio et al., 2013; Mao et al., 2022; Santos et al., 2019), *LNA* bacteria are associated with nutrient‐poor ecosystems (Mary et al., 2006; Wang et al., 2009). These features allow *HNA* bacteria to occupy more ecological niches (Hu et al., 2022), including microplastics (Dussud et al., 2018; Yang et al., 2020). *HNA* and *LNA* bacteria respond to environmental factors differently (Hu et al., 2023), making this functional classification useful to investigate the effects of stressors, microplastics in particular, on bacteria composition. In other words, observed changes in *HNA*:*LNA* ratios correspond to changes in bacterial community composition that may result from the presence of microplastics in the water column. Such changes in bacterial composition may affect interactions with phytoplankton communities, and eventually, ecosystem functions associated with these communities. This type of information is not provided by taxonomic approaches as the correspondence/correlation between taxonomic and functional groups within bacteria is not clear (Vila‐Costa et al., 2012).

Although the potential impacts of microplastics on marine microbial communities and their functioning are diverse, there is no consensus about what mechanism prevails over the others (Jacquin et al., 2019) and, consequently, no unequivocal evidence on the effects of microplastics on marine microbes (Galgani et al., 2019). Microplastics provide additional niche space, the so‐called *plastisphere* (Amaral‐Zettler et al., 2020; Sheridan et al., 2022), an artificial, hard, and persistent surface for microbial colonization. Evidence suggests that the plastisphere can alter marine microbial composition by hosting a different microbial community from that living in the free water (Dussud et al., 2018) and by increasing phytoplankton productivity and biomass (Yang et al., 2020). Microplastics can also influence photo‐inhibition, a phenomenon associated with the absorption of light by photosynthetic organisms in excess of that required for photosynthesis, which results in a reduced photosynthetic capacity. Thus, a higher phytoplankton biomass can result from restrictions in photo‐inhibition processes in the surface layer due to the presence of microplastics, which in turn allows for a more efficient use of light by photosynthetic organisms. Conversely, microplastics can reduce ecosystem productivity in a variety of ways, for example, by decreasing phytoplankton species diversity (Nava & Leoni, 2021), chlorophyll content (Cheng et al., 2021; Prata et al., 2018), and photosynthetic efficiency (Wright et al., 2013; Zhang et al., 2017). Microplastics also affect the interactions between bacteria and phytoplankton (e.g., mutualism or competition; Pomeroy et al., 2007) and bacteria‐driven nutrient cycling processes (e.g., increasing denitrification; Seeley et al., 2020), both considered important drivers of marine productivity. Together with the plethora of potential microplastic effects, another limitation of current research is that most of the observed effects of microplastics on microbial communities are obtained through laboratory experiments, where environmental conditions are highly controlled and biological communities are simplified. Therefore, it remains unknown whether such effects actually occur in natural marine ecosystems.

**FIGURE 1:**
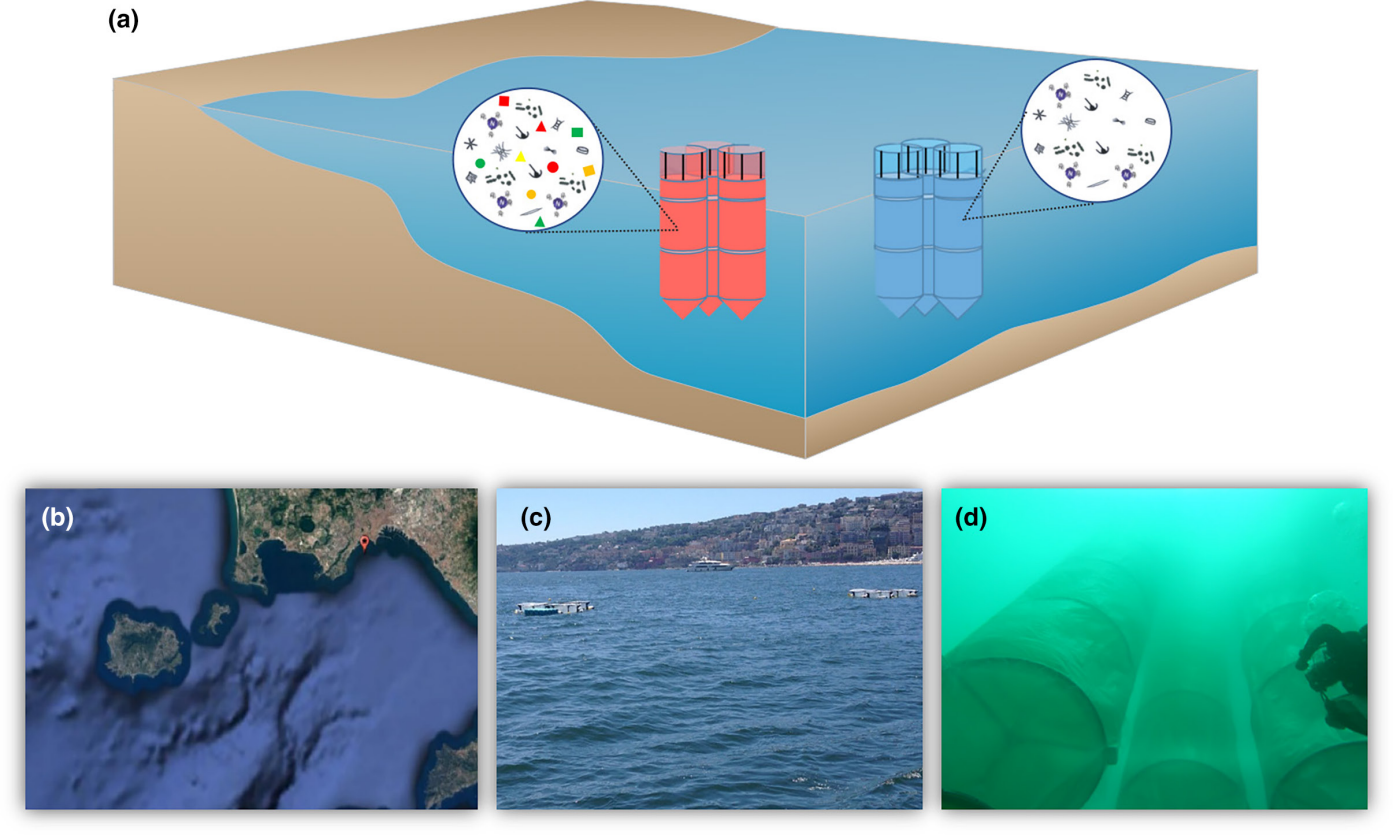
Mesocosm experimental design. (a) Schema of the six mesocosms. Blue and red cylindrical nets correspond to control treatments and treatments to which microplastics were added, respectively. Microbial communities composed of bacteria and phytoplankton were sampled within each net. Different particle colors within microplastic treatments indicate different types of microplastic. (b) Experimental location (Gulf of Naples, Mediterranean Sea). (c) View of the six mesocosms (two groups of three) from the sea surface. (d) Underwater picture of a three mesocosms group.

Here, we used an in situ mesocosms approach (Figure 1 and Figure S1) to investigate the microplastics‐induced changes in the structure and functioning of marine microbes, focusing on heterotrophic bacteria (hereafter bacteria) and phytoplankton communities. Our general goal is to test the hypothesis that microplastics affect both phytoplankton and bacterial communities as well as they affect the interactions between both communities, with cascading effects on marine productivity. More specifically, we test the following hypotheses:

Hypothesis 1 Microplastics enhance phytoplankton biomass as a result of light‐mediated responses—that is, photo‐inhibition.

Hypothesis 2 Microplastics addition changes bacterial community composition (*HNA* vs. *LNA* bacteria) by providing additional niche space that is differently colonized by different bacterial types.

Hypothesis 3 Changes in microbial assemblages will in turn affect their interactions, especially the competition between bacteria and phytoplankton communities.

Hypothesis 4 Because structure drives function in ecological systems (e.g., Hong et al., 2022), we expect that microplastic‐induced changes in bacteria and phytoplankton communities will ultimately influence marine productivity.

Hypothesis 5 Finally, microplastics differ in their size, shape, charge, and toxicity, and we expect that the response of microbial communities to their addition will vary across polymer types.

These hypotheses can be tested straightforwardly with the experimental mesocosm. However, because the effects of microplastics on ecosystem functioning can be direct or indirect (i.e., mediated by changes in community structure), we expand the analysis by using structural equations models (SEMs; Lefcheck, 2016) to capture such range of responses and provide a more mechanistic understanding of the effects of microplastics on different aspects of community structure and functioning.

## 2 MATERIALS AND METHODS

### 2.1 Mesocosms setup and experimental design

Six cylindrical nets (50 m^3^ each, 15 m deep) were set in the surface waters of a coastal Mediterranean area (Gulf of Naples). The six mesocosms were divided into two groups: the first (M1, M2, and M3, hereafter no microplastics group) was only treated with nutrient fertilization, while the second (M4, M5, and M6, hereafter microplastics group) with nutrient fertilization and microplastics addition. Therefore, the control treatment in our experiment has nutrients but not microplastics. Although a fully factorial experiment would require treatments without nutrients and microplastics, the use of nutrient‐only treatments as controls is justified in this case (see next section). The experimental design was similar to that reported by Giovagnetti et al. (2013) and Guieu et al. (2010). See Data S1 for further details on mesocosm setup and experimental design.

### 2.2 Nutrient fertilization

Nutrients (phosphate and silicate) were added to the six mesocosms to (i) prevent any potential depletion during the experiment and (ii) to boost microalgal growth. We measured the concentration of macronutrients—nitrate, phosphate, silicate, nitrite, and ammonia—during the setup of the experiment at sea, that is, before the addition of microplastics. The verified low concentration of phosphate and silicate resulted from the massive resource utilization during the past spring microalgal bloom. Input of phosphate was therefore determined to allow microalgae to bloom, while silicate was added to facilitate diatoms' growth, allowing competition between diatoms and non‐diatoms as it does occur at the onset of the spring bloom. Indeed, this situation simulates the in situ natural pre‐bloom condition and allows us to explore the effects of microplastics in a typical temperate coastal situation, that is, with no nutrient limitation, high biomass concentration, and enhanced biogeochemical fluxes. This is a typical and generic situation that can be used as a reference for the study of microplastic effects in coastal areas.

### 2.3 Microplastics preparation and addition

Enrichment with microplastics took place in three mesocosms. Microplastics were manufactured at the Polytechnic University of Marche according to Corinaldesi et al. (2021). Five different polymers were used: polystyrene (PS, density 1.04–1.09 g cm^−3^), polyethylene (PE, 0.89–0.95 g cm^−3^), polypropylene (PP, 0.85–0.92 g cm^−3^), polyvinylchloride (PVC, 1.16–1.41 g cm^−3^), and polyethylene terephthalate (PET, 1.34–1.41 g cm^−3^). These plastic types are among the most common in the marine environment. Plastic particles were finely ground and then size sieved under sterile conditions (Corinaldesi et al., 2021). Microplastics had a size range of 20–1000 μm and were brightly colored to facilitate their recognition among the different types (PS in pink, PE in blue, PP in yellow, PVC in orange, and PET in green; Corinaldesi et al., 2021). Also, colors allowed us to prevent any confusion with potential microplastics already present in the water column.

Once size separated, microplastics were mixed and stored in sterile glass jars. The final concentration of microplastics in the three mesocosms was 100 pieces L^−1^ (20 particles/polymer). This concentration was selected to represent the upper, yet realistic range of reported concentrations of microplastics in marine systems (e.g., Bucci et al., 2020; Corinaldesi et al., 2021; Kang et al., 2015; Pinto et al., 2019), likely underestimated by current sampling procedures (Hossain et al., 2019; Lindeque et al., 2020; Pinto et al., 2019). Indeed, greater concentrations were recently reported (Brandon et al., 2020) and experimentally used in in situ–simulated experiments (Galgani et al., 2019, 2023).

### 2.4 Variables sampled

To investigate the effects of microplastics on phytoplankton productivity mediated by bacterial and phytoplankton assemblages and their interaction, we measured the following variables: (1) chlorophyll *a* concentration (mg·m^−3^), as a proxy of phytoplankton biomass; (2) the proportion of high (*HNA*) versus low (*LNA*) nucleic acid concentration (*HNA*/[*HNA* + *LNA*]) as a proxy of the bacterial community structure; (3) the concentration of ammonium (NH_4_^+^), a key element of the nitrogen cycle essential for both bacteria and phytoplankton and used in photosynthesis; and (4) phytoplankton productivity, estimated using a proxy that reflects photosynthetic efficiency. These variables capture fundamental information on the structure and functioning of marine microbial communities. We also measured environmental variables relevant to test some of our hypotheses (e.g., light intensity within the water column), and the concentration of microplastics within the treatments.

#### 2.4.1 Phytoplankton biomass

We measured chlorophyll *a* concentration as a proxy of phytoplankton biomass. For this purpose, 50 mL of seawater was sampled daily at the three depths in the six mesocosms and stored in dark bottles until processing. Measurement of the relative fluorescence units was carried out with a fluorimeter model 10‐005R (Turner Designs), while the concentration (μg chl.*a* L^−1^) was obtained thanks to a calibration curve carried out with microalgal samples analyzed both with the fluorimeter (model 10‐005R) and with HPLC (Giovagnetti et al., 2013). We verified that the microplastics used did not interfere with the chl.*a* fluorescence, that is, they did not emit red light when blue light was provided.

#### 2.4.2 Bacterial community structure

Note that sequencing was not conducted to provide taxonomic information on bacterial organisms. This is because it is not clear whether *LNA* and *HNA* bacterial groups represent different bacterial fractions from the same bacterial species (Vila‐Costa et al., 2012), making taxonomical information less appropriate for assessing effects on ecological functions. Instead, we used a functional‐based approach that quantifies the compositional structure of the bacterial community as the proportion of high (*HNA*) versus low (*LNA*) nucleic acid concentration (*HNA*/[*HNA* + *LNA*]) bacteria. To quantify bacterial types, we used flow cytometry analysis. One mL seawater samples for heterotrophic prokaryote (HP) counts were fixed with a mix of glutaraldehyde (GL, 0.05% final concentration) and stored at −80°C until the analysis. Thawed samples were stained with SYBR green (Invitrogen) 10^−3^ dilution of stock solution for 15 min at room temperature. Cell concentrations were assessed using a FACScalibur flow cytometer (BD BioSciences Inc.) equipped with a 488 nm Ar laser and standard set of optical filters. FCS Express software was used for analyzing the data and HP was discriminated from other particles on the basis of scatter and green fluorescence from SYBR green (Balestra et al., 2011). Two subpopulations were discriminated based on their relative green fluorescence (as a proxy of DNA content) and denominated low nucleic acid (*LNA*) and high nucleic acid (*HNA*), respectively. The cut‐off of the particle size analyzed by flow cytometry was around 5 μm, that is, much lower than the microplastic size range (20–1000 μm). Indeed, the presence of microplastic pieces in the cytograms of the analyzed samples was excluded.

We also measured extracellular β‐glucosidase activity as a complementary metric associated with bacterial community structure. Extracellular β‐glucosidase activities were determined in seawater samples through the analysis of the cleavage rates of the artificial fluorogenic substrate 4‐methylumbelliferyl MUF‐b‐d‐glucopyranoside under saturating substrate concentrations (Danovaro et al., 2005). Seawater samples were incubated in the dark at the in situ temperature, then analyzed fluorometrically (365 nm excitation, 455 nm emission) immediately after addition of the substrate and following incubation. The detected increase in fluorescence was converted into activity using standard curves with 4‐methylumbelliferone (Danovaro et al., 2005).

#### 2.4.3 Marine productivity

We focused on phytoplankton productivity, and it was measured as the maximum quantum yield of primary photochemistry, which is the ratio between variable and maxima fluorescence (*F*_v_ */F*_m_) and reflects photosynthetic efficiency (Gorbunov & Falkowski, 2022). Variable fluorescence was measured daily on freshly collected samples, with a Phyto‐PAM (Heinz Walz GmbH), following the procedure successfully developed during a previous mesocosms experiment (Giovagnetti et al., 2013). Fifty mL of seawater was sampled daily at each depth and stored in the dark. After 30 min in dark, a 3 mL aliquot was used for measurements of quantum yield of fluorescence. The quantum yield of fluorescence for the 15 min dark‐adapted samples (*F*_v_ */F*_m_, with *F*_v_ = *F*_m_ *– F*_o_) was determined through the measurements of the minimum fluorescence level *F*_o_ and the maximum fluorescence level *F*_m_. The latter corresponds to the maximum fluorescence measured after a saturation pulse of bright red light (655 nm, 2400 μmol photons·m^−2^ s^−1^) applied during 450 ms. The pulse was saturating since the increase in its duration did not increase the fluorescence yield in any of the analyzed samples.

#### 2.4.4 Environmental variables

Temperature and light intensity inside the mesocosms were recorded using HOBO Pendant® data loggers (Onset Computer Corporation). Loggers were placed at three depths in each mesocosm: 0.5, 4.5, and 9.5 m depth. The data loggers provided both light and temperature measurements every 5 min during the experiment duration. Light intensity was provided in LUX, which was converted to photosynthetic photon flux (PPF) by multiplying LUX values by a factor of 0.0158 (Thimijan & Heins, 1983). Data acquired by loggers were then compared to light intensity measurements in air to verify that internal structures of mesocosms did not disturb the light penetration.

#### 2.4.5 Nutrient concentration

To determine the concentration of macronutrients, 20 mL of seawater was sampled daily at 0.5, 4.5, and 9.5 m depth in each mesocosm and stored at −20°C. Concentrations of nitrate (NO_3_^−^), nitrite (NO_2_^−^), ammonium (NH_4_^+^), silicic acid (SiO_4_^−^), and phosphate (PO_4_^3−^) were determined with an autoanalyzer using the colorimetric procedure described by Grasshoff et al. (1983).

#### 2.4.6 Microplastic concentration

One liter of seawater was sampled daily, at 0.5, 4.5, and 9.5 m depths in all mesocosms. Once in the laboratory, water samples were filtered onto 10 μm nylon mesh filters. Then, filters were folded and stored at −20°C until processing. To remove the organic matter without damaging the microplastics, filters were placed in glass Petri dishes containing 20 mL of 30% hydrogen peroxide and left at room temperature for 1 h. Filters were therefore rinsed with distilled water and the content of the Petri dishes was re‐filtered onto Nuclepore® Track‐Etch membrane filters (Corning, porosity of 10 μm, diameter 25 mm), and placed on microscope slides for counting. Counts were done with a Leica M165C stereoscope with a light source positioned above the filter to facilitate the identification of the different microplastic types by their color. For each filter, all five types of added microplastic polymers were counted and two pictures were taken for further count verification. The analyses performed to determine microplastic concentrations in seawater were done using glass or metal laboratory equipment to minimize the risk of contamination from external sources.

### 2.5 Statistical analyses

For each variable, the normalized daily anomaly (*NDA*; Galgani et al., 2014; Seeley et al., 2020) was computed at each depth and for each mesocosm. The *NDA* represented the daily difference between the variable value measured in one mesocosm and one depth and the mean of the overall variable value at this depth for all the mesocosms and all the sampling times. The latter was used to normalize the difference. Then, we calculated the three depths mean and SD of the daily normalized anomaly. Differences in normalized daily anomalies between control and treated mesocosms were tested by Mann–Whitney with a significance level of *p* < .05.

We first performed simple regression analyses of bacterial composition (*HNA*/[*HNA + LNA*]), phytoplankton biomass (chlorophyll *a*), NH_4_^+^ concentration, and the maximum quantum yield of primary photochemistry reflecting the photosynthetic efficiency (*F*_v_ *F*_m_), as a function of microplastics concentration. Due to the diversity of mechanisms by which microplastics can potentially influence microbial communities and marine productivity, we then used structural equation models (SEMs; Lefcheck, 2016), which provide a more mechanistic understanding of the direct and indirect effects among microplastics and the structure and functioning of bacteria and phytoplankton communities. The focus of the SEM analysis is on disentangling the potential mechanisms driving changes in marine productivity under the presence of microplastics. We built SEMs for total microplastics as well as for each microplastic type individually (polystyrene [PS], polypropylene [PP], polyethylene terephthalate [PET], polyvinyl chloride [PVC], and polyethylene [PE]). We allowed microplastics to affect bacterial composition (*HNA*/[*HNA + LNA*]), phytoplankton biomass (chlorophyll *a*), NH_4_^+^ concentration, and the maximum quantum yield of primary photochemistry reflecting the photosynthetic efficiency (*F*_v_ *F*_m_). Bacterial composition could affect phytoplankton biomass via competition, and NH_4_^+^, whereas phytoplankton biomass and NH_4_^+^ could affect photosynthetic efficiency.

The goodness of fit of *piecewise* SEMs is tested using the directed separation (*d‐sep*) test proposed by Shipley (2013). In the SEM context, it is a test of the conditional independence claims implied by the model structure. The significance of any given independence claim is measured by its *p‐*value (local estimation), with its corresponding *R* ^2^. In mixed‐effects models, both marginal (concerned about *fixed* effects only) and conditional (concerned about both *fixed* and *random* effects) *R* ^2^s are provided. We used mixed‐effects models with mesocosm ID as random factor. The test of directed separation is conducted by combining all *p*‐values across the basis set in a test statistic (Fisher's *C*; global estimation). If there is insufficient evidence to reject any of the conditional independence claims implied by the hypothesized structure, then the data are said to support this model of causality. Furthermore, the standardized coefficients of the models fitted to the data inform us about the relationships between variables, that is, the size and sign of the causal effect of one variable on another. The Akaike information criterion (AIC) is used to select the causal model that best fits the data.

We implemented SEM analysis using the R package *PiecewiseSEM* (version 2.2.0; Lefcheck et al., 2018), using linear mixed‐effects models (*nlme* package, version 3.1–155) to model the relationships between variables, with mesocosm ID as random effect. Since depth made no qualitative difference in the SEM results (did not change the sign of significant links; Figure S2), we decided to aggregate over the three depths. This aggregation effectively increased the number of observations per mesocosm. Prior to fitting the models, variables were checked and transformed, if necessary, to ensure linearity and normality. Specifically, all variables, except for NH_4_^+^, met normality criteria. NH_4_^+^ was log transformed to fit normality. We then fitted the fully connected SEM model and, following Shipley's methodology, iteratively removed the least significant links (highest *p*) to obtain the simplest causal model consistent with the data. On each iteration, the SEM was refitted with one link removed, and the AIC_c_ and Fisher's *p* were tested. As a result, the direct link between microplastics and photosynthetic efficiency was removed from the model structure. All the remaining hypothesized relationships were statistically supported by the data (*p* < .05), and no missing paths were identified.

## 3 RESULTS AND DISCUSSION

**FIGURE 2:**
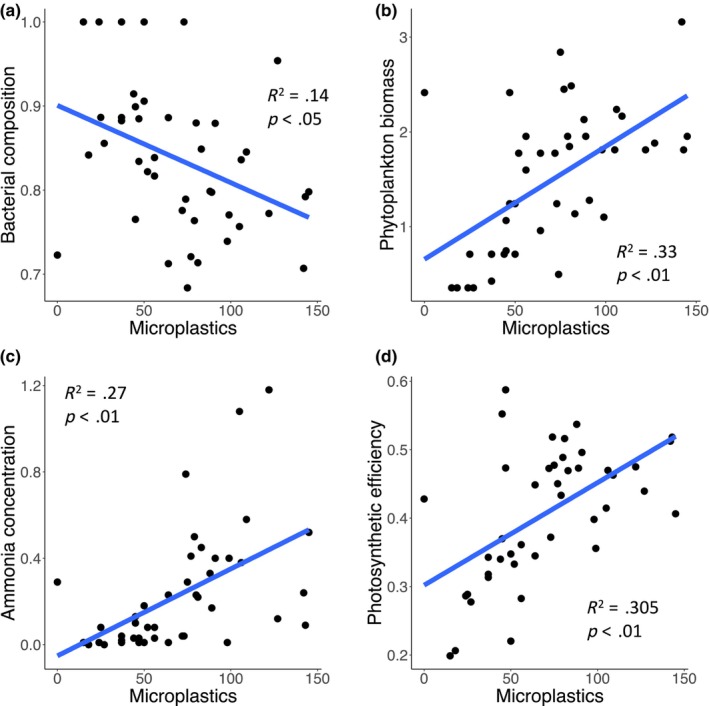
The effect of microplastics on several aspects of the structure and functioning of marine microbial communities. Plots show linear regressions between microplastic concentrations and structural and functional variables. Bacterial composition is represented by the proportion of high (*HNA*) versus low (*LNA*) nucleic acid concentration (*HNA*/[*HNA* + *LNA*]) as a proxy of the bacterial community structure (a). Chlorophyll *a* concentration (mg·m^−3^) was used as a proxy of phytoplankton biomass (b). Ammonia concentration (NH_4_^+^), a key element ofthe nitrogen cycle essential for bacteria and phytoplankton and used in photosynthesis (c). Productivity is represented by photosynthetic efficiency, measured as the ratio between variable and maxima fluorescence (*F*_v_ */F*_m_) (d).

The mesocosm experiment showed consistent effects of microplastics on marine microbial communities and their functioning (Figure 2). Our analyses gave support to microplastics‐induced rises in phytoplankton biomass (Figure 2b, *R* ^2^ = .33, *p* < .01), ammonia concentration (Figure 2c, *R* ^2^ = .27, *p* < .01), and marine productivity (Figure 2d, *R* ^2^ = .31, *p* < .05). Also, microplastics changed bacterial community composition by significantly reducing the proportion of *HNA* bacteria in the water column (Figure 2a, *R* ^2^ = .14, *p* < .05). These results confirm our expectations that the presence of microplastics in the ocean influence key aspects of microbial community structure and functioning.

### 3.1 Phytoplankton biomass (Hypothesis 1)

**FIGURE 3:**
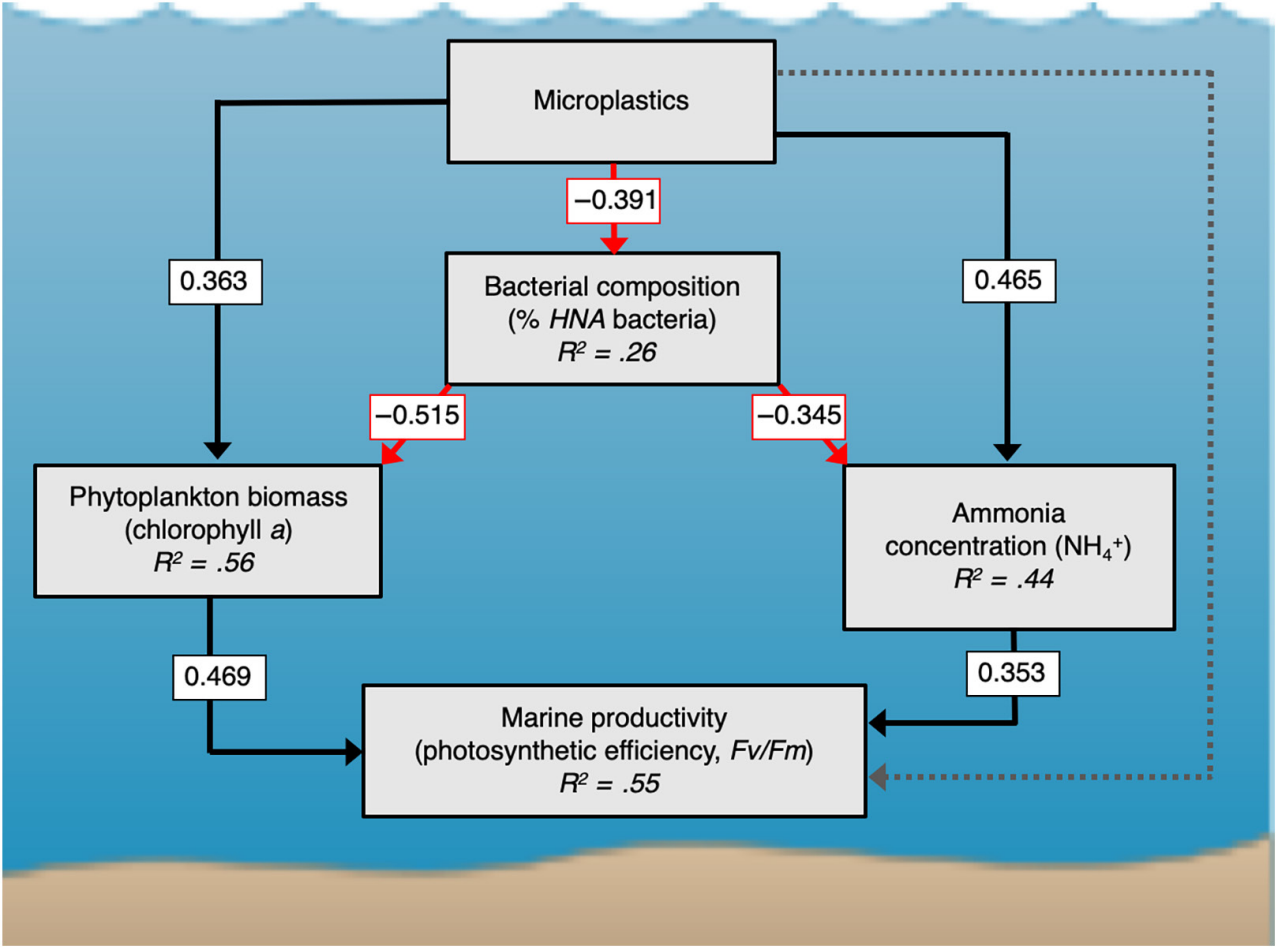
Structural equation model exploring the effects of microplastics on marine productivity. Productivity is represented by photosynthetic efficiency, which is measured as *F*_v_ */F*_m_. Black and red solid arrows denote positive and negative associations, respectively. Dashed paths indicate no detectable influence of the driver (*p* ≥ .05). *Fisher's C* = 3.738; *df* = 6; *p* = .712; AIC_c_ = 14.796. Numbers in boxes represent the standardized coefficient of each path. *R* ^2^s are reported as the conditional *R* ^2^ based on the variance of both the fixed and random effects. Individual models for different microplastic types are presented in Table 1.

**FIGURE 4:**
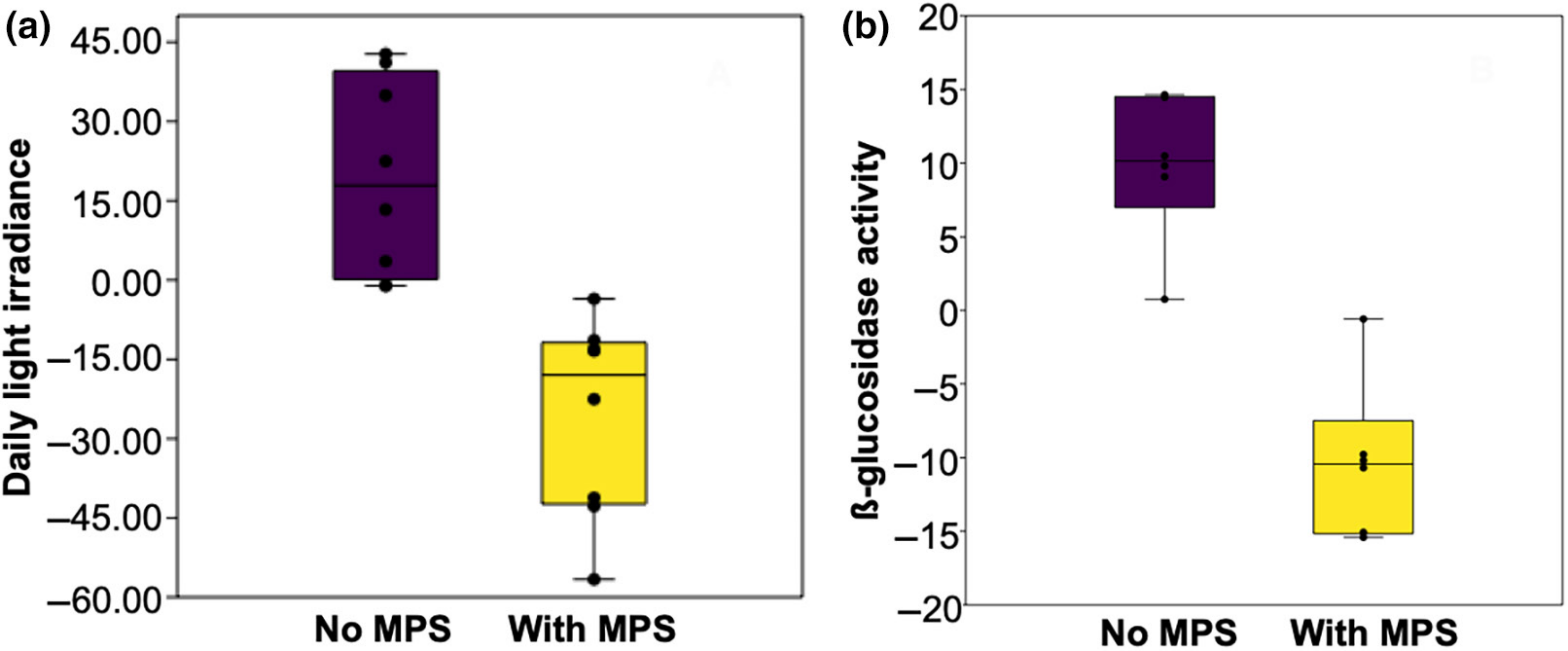
Microplastics' (MPS) effects on light conditions and β‐glucosidase activity. (a) Light conditions (daily light irradiance) with and without microplastics measured as anomalies of the daily light irradiance at 14 h00 (local time) (Mann–Whitney test, *N* = 8, *p* = .0009). (b) Daily β‐glucosidase activity with and without microplastics (Mann–Whitney test, *N* = 6; *p* = .005).

Our results from SEMs showed that the effects of microplastics on marine productivity were all indirect and mediated by changes in the bacterial and phytoplankton assemblages (Figure 3, Fisher's *C* = 3.738, *p* = .712, *df* = 6). On one hand, microplastics directly enhanced phytoplankton biomass in the water column (0.363, *p* < .005) and, consequently, photosynthetic efficiency (Figure 3, standardized coefficient [SC] = 0.469, *p* < .001). According to our first hypothesis, this increase in phytoplankton biomass is primarily driven by the reduction of light intensity in the water column due to the presence of microplastics (Figure 4a) that in turn limits photo‐inhibition in the surface layer, enabling more efficient use of light (Hypothesis 1; Figure 3, SC = 0.469, *p* < .001).

### 3.2 Bacterial community composition (Hypothesis 2)

On the other hand, microplastics altered bacterial community composition by reducing free‐living *HNA* bacteria relative to *LNA* bacteria (Hypothesis 2; Figures 2a and 3), which triggered a number of indirect effects that we detail later. The plastisphere is a carbon‐ and nitrogen‐rich microenvironment (Fauvelle et al., 2021; Romera‐Castillo et al., 2018), particularly suitable for the fast‐growing *HNA* bacteria community. As expected from our second hypothesis, this preferential colonization of microplastics by *HNA* bacteria explains the compositional changes observed in the water column, with an increase in the less active *LNA* bacteria and a lower proportion of free‐living *HNA* bacteria (Figure 3, SC = −0.391, *p* < .01). This shift in the bacterial assemblage is responsible for the observed decrease in β‐glucosidase activity (Figure 4b). Therefore, the relative abundance of bacteria types known to be regulated by environmental factors such as water temperature or nutrient concentrations (Pradeep Ram et al., 2020; Liu et al., 2016; Moran et al., 2015) is also mediated by the presence of microplastics. As we did not use sequencing data, no taxonomic information was available. However, it is not clear whether *LNA* and *HNA* bacterial groups represent different bacterial fractions from the same bacterial species (Vila‐Costa et al., 2012), and this renders taxonomical information less useful if we aim to assess effects of microplastics on ecological functions. Therefore, for the specific purpose of this study, using a functional‐based instead of a taxonomic‐based approach is more informative to quantify bacterial composition.

### 3.3 Interactions between bacteria and phytoplankton communities (Hypothesis 3)

The observed changes in bacterial composition affect marine productivity through two additional mechanisms. The first mechanism is the competitive release of phytoplankton. We observed *LNA* bacteria increased in abundance in the water column relative to *HNA* bacteria. As *LNA* bacteria have a lower metabolic rate than *HNA* bacteria (Hu et al., 2020; Liu et al., 2016; Moran et al., 2015), they are less efficient in exploiting environmental resources (nutrients) and exert a reduced competition on phytoplankton. This competitive release allows phytoplankton to increase in biomass, which supports our third hypothesis (Hypothesis 3; Figure 3, SC = −0.515, *p* < .01). Thus, microplastics promote phytoplankton biomass directly, by providing a more suitable light environment (Hypothesis 2), and, indirectly, by reducing the competition with bacteria (Hypothesis 3).

### 3.4 Marine productivity (Hypothesis 4)

A second mechanism derived from the altered bacterial composition is mediated by the cycling of NH_4_^+^, which increases its concentration in the water column. Whereas *LNA* bacteria in the water column did not significantly uptake NH_4_^+^ and favored its persistence in the water (Figure 3, SC = −0.345, *p* < .01), *HNA* bacteria remineralize the nitrogen associated with the plastisphere, releasing it as NH_4_^+^ into the surrounding environment (Figure 3, SC = 0.465, *p* < .001). These results agree with recent findings reporting an opposite trend between *HNA*:*LNA* ratios and NH_4_^+^ (Hu et al., 2023), are consistent with recent observations in sediment microbial communities, where microplastic contamination resulted in an accumulation of NH_4_^+^ following changes in community composition (Seeley et al., 2020), and complement recent observations of microplastics being a potential source of N_2_O emission (Su et al., 2022). SEMs show that the pathway involving *HNA* bacteria is stronger, with the NH_4_^+^ concentration increase mainly driven by the activity of microplastics‐attached *HNA* bacteria (total effect size [TES] = 0.465 vs. −0.135). NH_4_^+^ is efficiently used as nitrogen source by phytoplankton in the process of photosynthesis (Ruan & Giordano, 2017) so that changes in microplastics‐attached versus free‐living bacteria assemblages indirectly enhance marine photosynthetic productivity (Hypothesis 4: Figure 3, SC = 0.353, *p* < .01). These results confirm that microplastics are biologically active rather than inert material, and suggest that the accumulation of NH_4_^+^ is a consequence of the microplastics‐induced partition of the bacteria community between free‐living (*LNA*) and microplastic‐attached (*HNA*) assemblages. Therefore, in line with our fourth hypothesis, the effects of microplastics on marine productivity are both direct, by increasing phytoplankton biomass, and indirect, by releasing NH_4_^+^ which in turn results from changes in the marine microbial composition.

### 3.5 Response to different microplastic types (Hypothesis 5)

Contrary to our expectations (Hypothesis 5), the effects of microplastics on marine productivity were consistent for the different microplastic types considered, despite size, shape, and surface physicochemical properties of microplastics do affect bacteria colonization (Cheng et al., 2021; Hossain et al., 2019; Pinto et al., 2019). Our results suggest that the responses of bacterial and phytoplankton communities to microplastic pollution are common among the microplastic types. NH_4_^+^‐mediated processes (Table 1, TES = [0.098–0.226]) and light‐mediated responses (Table 1, TES = [0.114–0.239]) are the strongest mechanisms increasing marine productivity, with reduced competition between bacteria and phytoplankton playing a secondary, yet significant, role (TES = [0.059–0.105]; Table 1). However, models separately run for individual microplastics suggested some microplastic type‐specific responses. For example, our analysis suggested that the release of NH_4_^+^ is modulated by the capacity of microplastics to attract and attach bacteria, the latter being mediated by the physical and chemical microplastic properties. Also, PE has a negative charge in seawater that probably hinders bacteria attachment (Hossain et al., 2019), and this may explain why, although the best model for PE includes the link between *HNA* and microplastics, there is no evidence to support this relationship (*p* = .245). Although our study targeted five different microplastic types that are commonly found in the ocean, we cannot rule out the possibility that other types of microplastics could produce different effects.

**TABLE 1:** Microplastic effects on marine microbial processes.

Effects/mechanisms	Total plastics	Plastic type				
		PS	PP	PET	PVC	PE
NH_4_^+^ mediated	0.212	0.226	0.191	0.098	0.216	0.185
NH_4_^+^ release (*HNA* bacteria on microplastics)	0.164	0.180	0.135	0.078	0.167	0.156
NH_4_^+^ not uptake (*LNA* bacteria in free water)	0.048	0.046*	0.056	0.020	0.049	0.029*
Competition release (lower competition of phytoplankton with bacteria)	0.094	0.103	0.102	0.059	0.105	0.051*
Light‐mediated responses	0.170	0.239	0.200	0.114	0.191	0.170

*Note*: The effects of microplastics on photosynthetic efficiency are consistent across different microplastic types. Numbers in the table indicate standardized coefficients (SC) of each individual model quantified as total effect sizes (TES).

Abbreviations: Plastic type: PE, polyethylene; PET, polyethylene terephthalate; PP, polypropylene; PS, polystyrene; PVC, polyvinyl chloride (*indicates not sgnificant paths (*p*<0.05)).

## 4 CONCLUSIONS

Our results mimic the effects of microplastics on highly productive systems, like typical microalgal spring or autumn blooms in temperate coastal systems. In this study, the bloom condition was triggered by nutrient fertilization. Alternative ecological scenarios are possible, where our results may or may not hold. For example, in experiments with no nutrient addition, biological fluxes, the carbon cycle, and phytoplankton growth will be smaller than those observed in our experiment. This latter system represents more typical coastal summer conditions characterized by smaller organisms that require less energy and heterotrophic community (D'Alelio et al., 2016; Durrieu de Madron et al., 2011).

Microplastics concentrations in the marine environment are expected to further increase in the coming decades (Ruan & Giordano 2017), making them a global change driver with potential impacts on many ecosystems. The effects of microplastics on ecosystem functioning are starting to be revealed, as research has begun to shift from a more ecotoxicological view focusing on individual organisms, to fully embrace a community and ecosystem perspective (Hossain et al., 2019; Ingraffia et al., 2022; Legendre et al., 2015; Mao et al., 2018; Sheridan et al., 2022; Wang et al., 2022). This study confirms our hypotheses that experimental addition of microplastics increases phytoplankton biomass and shifts bacterial assemblages' composition, modifying the interactions between bacteria and phytoplankton and the amount of ammonia in the water column, which ultimately favors photosynthetic efficiency. Contrary to our expectations, microplastics' identity does not seem to strongly influence the observed responses, at least for the range of microplastics considered. By providing a carbon‐rich substrate for *HNA* bacteria, microplastics could enhance microbial respiration and the production of bacteria‐derived dissolved organic carbon. Consequently, together with other global change factors such as changes in temperature and nutrient concentrations (Hossain et al., 2019), the presence of microplastics, by adding both C and N into the ecosystem, may alter the balance between photosynthesis and respiration, with potential effects on microbial carbon sequestration (Legendre et al., 2015). The application and expansion of our findings to larger spatial and temporal scales will deepen our knowledge of the effects of microplastics in marine ecosystems, and to mitigate their impacts (Amaral‐Zettler et al., 2020; Galloway et al., 2017).

## AUTHOR CONTRIBUTIONS

**Daniel Montoya:** Conceptualization (equal); formal analysis (equal); investigation (equal); methodology (equal); software (lead); visualization (lead); writing – original draft (lead); writing – review and editing (equal). **Eugenio Rastelli:** Data curation (equal); formal analysis (equal); investigation (equal); methodology (equal); resources (equal); software (equal); writing – review and editing (equal). **Raffaella Casotti:** Data curation (equal); formal analysis (equal); writing – review and editing (equal). **Vincenzo Manna:** Data curation (equal); formal analysis (equal); writing – review and editing (equal). **Anna Chiara Trano:** Data curation (equal); formal analysis (equal); writing – review and editing (equal). **Cecilia Balestra:** Data curation (equal); formal analysis (equal); writing – review and editing (equal). **Chiara Santinelli:** Data curation (equal); formal analysis (equal); writing – review and editing (equal). **Maria Saggiomo:** Data curation (equal); formal analysis (equal); writing – review and editing (equal). **Clementina Sansone:** Data curation (equal); formal analysis (equal); writing – review and editing (equal). **Cinzia Corinaldesi:** Data curation (equal); formal analysis (equal); writing – review and editing (equal). **Jose M. Montoya:** Conceptualization (equal); investigation (equal); methodology (equal); writing – original draft (equal); writing – review and editing (equal). **Christophe Brunet:** Conceptualization (equal); data curation (equal); formal analysis (equal); funding acquisition (equal); investigation (equal); methodology (equal); project administration (lead); resources (equal); supervision (equal); writing – original draft (equal); writing – review and editing (equal).

## CONFLICT OF INTEREST STATEMENT

The authors declare no competing interests.

## Supporting information


Data S1:

## Data Availability

Data and metadata are provided as Supplementary Material in four files (Dataset.xlxs, Light.xlsx, Microplastics code.rtf, and README.rtf). These files will be available in the Zenodo data repository (https://zenodo.org/records/10617960; doi: 10.5281/zenodo.10617959).
