# Supplementary material for: Microplastics alter the functioning of marine microbial ecosystems
Source: Ecol Evol. 2024 Nov 14;14(11):e70041. doi: 10.1002/ece3.70041 (PMC11564128; doi:10.1002/ece3.70041)
Supplement: Supplementary file 1 — Data S1: [file ECE3-14-e70041-s001.zip › Metadata.rtf]

# Microplastics dataset---This repository includes the data and code used in Montoyal et al (ms number). More specifically:- Dataset.xlsx includes data on MPS, bacteria and phytoplankton data, environmental variables, and ocean productivity- ‘Microplastics code’ refers to the programming code used for the statistical analyses and plot construction using R software.## Description of the data and file structureThe data is provided with variables as columns. The abbreviations are explained below:id = sampling mesocosm and timeday = sampling daytemp = temperature (celsius degrees)depth = ocean depth (meters)mesocosm = mesocosm IDplast_tot = total concentration of microplastics (g cm-3)plast_ps = concentration of polystyrene (g cm-3)plast_pp = concentration of polypropylene (g cm-3)plast_pet = concentration of polyethylene terephthalate (g cm-3)plast_pvc = concentration of polyvinyl chloride (g cm-3)plast_pe = concentration of polyethylene (g cm-3)ammonium = Concentration of ammonium (NH4+) (mg m-3)hna = high nucleic acid concentration bacteria (% over total bacteria)lna = low-nuceic acid concentrationbacteria (% over total bacteria)chla_fluo = phytoplankton biomass, measured as chlorophyll a concentration (mg m-3)fvfm = photosynthetic efficiency, measured as the ratio between variable and maxima fluorescence (Fv/Fm) ## Sharing/Access informationNot relevant## Code/Software‘Microplastics code’ refers to the programming code used for the statistical analyses and plot construction using R software.
