# Supplementary material for: Microplastics alter the functioning of marine microbial ecosystems
Source: Ecol Evol. 2024 Nov 14;14(11):e70041. doi: 10.1002/ece3.70041 (PMC11564128; doi:10.1002/ece3.70041)
Supplement: Supplementary file 1 — Data S1: [file ECE3-14-e70041-s001.zip › Microplastics code.rtf]

######## Code for #### Load librarieslibrary(piecewiseSEM)library(nlme)library(tidyverse)#### Read datasetplastics <- readxl::read_excel("/Users/Daniel/Desktop/Dataset.xlsx")#Calculate bacterial compositionhnalna=plastics$hna/(plastics$hna+plastics$lna)plastics=cbind(plastics,hnalna)# Subset dataset (choose plastic type, for example). Below, total microplastics is chosen as an example:sub_vars <- c("day", "id", "plast_tot", "hna", "lna", "hnalna", "ammonium", "chla_fluo", "fvfm", "mesocosm", "temp", "depth")# Omit NaN valuesplastics_sub <- plastics %>% select(sub_vars) %>% na.omit()#### SIMPLE REGRESSIONS PRIOR TO SEMs# Bacterial composition ~ F(Microplastics)ggplot(plastics_sub, aes(x=plast_tot, y=hnalna)) + geom_point(size = 4) + geom_smooth(method=lm, se=FALSE, fullrange=TRUE, size = 3) + theme_classic() + ggtitle("") + xlab("Microplastics") + ylab("Bacterial composition") + theme(axis.title.x = element_text(size=25), axis.title.y = element_text(size=25), axis.text.x  = element_text(size = 20), axis.text.y  = element_text(size = 20), plot.margin = margin(t = 30,  # Top margin                                                                                                                                                                                                                                                                                                                                                                                                                      r = 50,  # Right margin                                                                                                                                                                                                                                                                                                                                                                                                                      b = 20,  # Bottom margin                                                                                                                                                                                                                                                                                                                                                                                                                      l = 10)) # Left marginsummary(lm(plastics_sub$hnalna~plastics_sub$plast_tot))# Phytoplankton biomass ~ F(Microplastics)ggplot(plastics_sub, aes(x=plast_tot, y=chla_fluo)) + geom_point(size = 4) + geom_smooth(method=lm, se=FALSE, fullrange=TRUE, size = 3) + theme_classic() + ggtitle("") + xlab("Microplastics") + ylab("Phytoplankton biomass") + theme(axis.title.x = element_text(size=25), axis.title.y = element_text(size=25), axis.text.x  = element_text(size = 20), axis.text.y  = element_text(size = 20), plot.margin = margin(t = 30,  # Top margin                                                                                                                                                                                                                                                                                                                                                                                                                         r = 50,  # Right margin                                                                                                                                                                                                                                                                                                                                                                                                                         b = 20,  # Bottom margin                                                                                                                                                                                                                                                                                                                                                                                                                         l = 10)) # Left marginsummary(lm(plastics_sub$chla_fluo~plastics_sub$plast_tot))# Ammonia concentration ~ F(Microplastics)ggplot(plastics_sub, aes(x=plast_tot, y=ammonium)) + geom_point(size = 4) + geom_smooth(method=lm, se=FALSE, fullrange=TRUE, size = 3) + theme_classic() + ggtitle("") + xlab("Microplastics") + ylab("Ammonia concentration") + theme(axis.title.x = element_text(size=25), axis.title.y = element_text(size=25), axis.text.x  = element_text(size = 20), axis.text.y  = element_text(size = 20), plot.margin = margin(t = 30,  # Top margin                                                                                                                                                                                                                                                                                                                                                                                                                        r = 50,  # Right margin                                                                                                                                                                                                                                                                                                                                                                                                                        b = 20,  # Bottom margin                                                                                                                                                                                                                                                                                                                                                                                                                        l = 10)) # Left marginsummary(lm(plastics_sub$ammonium~plastics_sub$plast_tot))# Photosynthetic efficiency ~ F(Microplastics)ggplot(plastics_sub, aes(x=plast_tot, y=fvfm)) + geom_point(size = 4) + geom_smooth(method=lm, se=FALSE, fullrange=TRUE, size = 3) + theme_classic() + ggtitle("") + xlab("Microplastics") + ylab("Photosynthetic efficiency") + theme(axis.title.x = element_text(size=25), axis.title.y = element_text(size=25), axis.text.x  = element_text(size = 20), axis.text.y  = element_text(size = 20), plot.margin = margin(t = 30,  # Top margin                                                                                                                                                                                                                                                                                                                                                                                                                        r = 50,  # Right margin                                                                                                                                                                                                                                                                                                                                                                                                                        b = 20,  # Bottom margin                                                                                                                                                                                                                                                                                                                                                                                                                        l = 10)) # Left marginsummary(lm(plastics_sub$fvfm~plastics_sub$plast_tot))######## STRUCTURAL EQUATION MODELS (SEMs) #############3# 5-VARIABLE MODEL (plastics, phytoplankton (structure & function), bacteria, NH4)#### Tranformation of variables to meet normality criteria # Check for normalitydata=as.data.frame(plastics_sub)shapiro = matrix(nrow=length(data), ncol=1, 0)rownames(shapiro)=names(data)colnames(shapiro)=c("p_value")# Choose columns/variablesfor(i in c(3,6:9)) {  s=shapiro.test(data[,i])  shapiro[i]=as.numeric(s[2])}shapiro=as.data.frame(shapiro)which(shapiro>0.05)# Transformation:plastics_sub$ammonium <- log10(plastics_sub$ammonium + 0.01)plastics_sub$plast_pe <- log10(plastics_sub$plast_pe)plastics_sub$plast_pp <- log10(plastics_sub$plast_pp)plastics_sub$plast_ps <- sqrt(plastics_sub$plast_ps)plastics_sub$plast_pet <- sqrt(plastics_sub$plast_pet)# Final model (hypotheses as fixed factors, mesocosm as a random factor)mod1 = lme(hnalna ~ plast_tot, random = ~ 1 | mesocosm, na.action = na.omit,plastics_sub)mod2 = lme(chla_fluo ~ plast_tot + hnalna, random = ~ 1 | mesocosm, na.action = na.omit,plastics_sub)mod3 = lme(ammonium ~ hnalna + plast_tot, random = ~ 1 | mesocosm, na.action = na.omit,plastics_sub)mod4 = lme(fvfm ~ chla_fluo + ammonium, random = ~ 1 | mesocosm, na.action = na.omit, plastics_sub)global_model <- psem(  mod1,  mod2,  mod3,  mod4)summary(global_model)plot(global_model)summary(global_model)$AIC$AICc
