# Supplementary material for: Microplastics alter the functioning of marine microbial ecosystems
Source: Ecol Evol. 2024 Nov 14;14(11):e70041. doi: 10.1002/ece3.70041 (PMC11564128; doi:10.1002/ece3.70041)
Supplement: Supplementary file 1 — Data S1: [file ECE3-14-e70041-s001.zip › Supporting_Information.docx]

# **Supplementary Information**

# Section S2. Supplementary Figures and Tables

Figure S1 | Photos of the mesocosms deployment at sea. **(a)** Mesocosms on the shore waiting for towing boat. **(b)** View of a group of three mesocosms from the seafloor. **(c)** Photo of the junction between the bottom cone and the plastic bag. **(d)** Photo showing the two groups of three mesocosms after all the deployment operation.

**
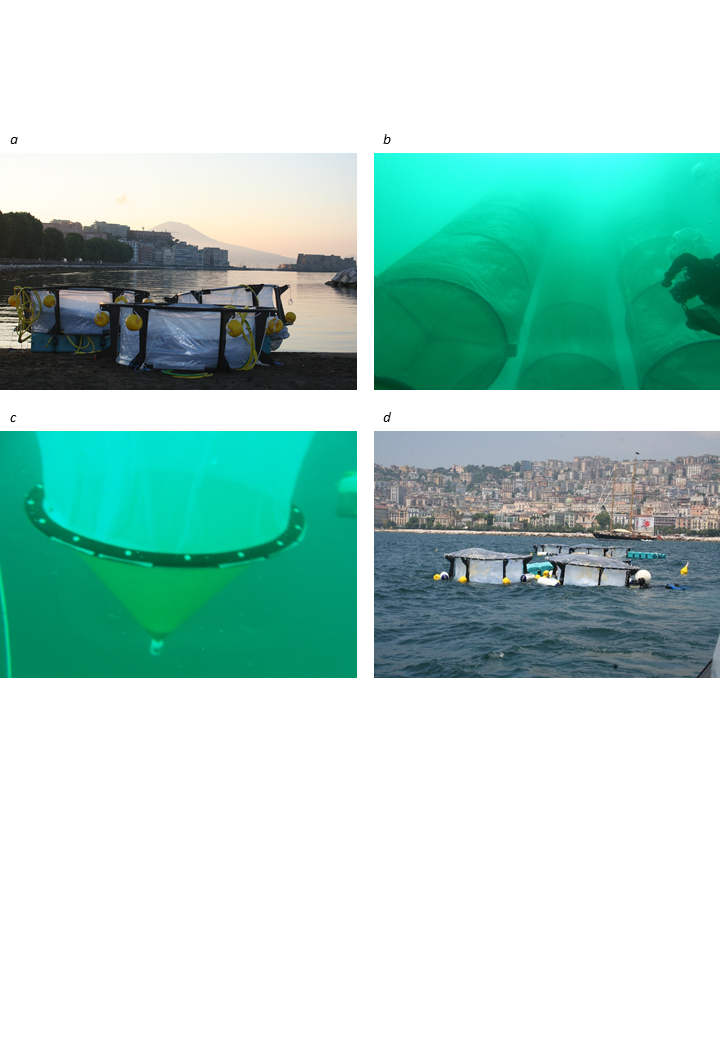
**

Figure S2 | Bivariate plots at different depths. Bivariate plots of the relevant variables in structural equation models as a function of total microplastics for different depths. Bivariate relationships were similar at the three different depths.

**
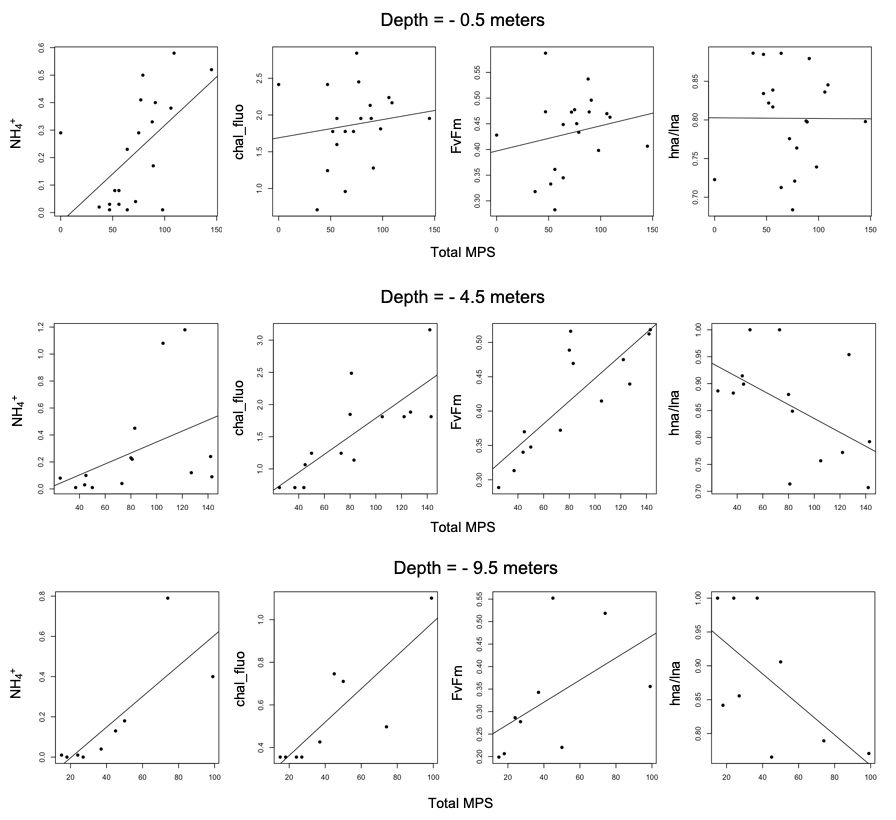
**
